# The Effect of Visual Function on the Batting Performance of Professional Baseball Players

**DOI:** 10.1038/s41598-019-52546-2

**Published:** 2019-11-14

**Authors:** Daniel M. Laby, David G. Kirschen, Usha Govindarajulu, Paul DeLand

**Affiliations:** 1 SportsVisionNYC, New York, NY USA; 2 Southern California College of Optometry, Marshall Ketchum University, Fullerton, CA USA; 3 Jules Stein Eye Institute, University of California at Los Angeles, Los Angeles, CA USA; 4 College of Optometry, State University of New York, New York, NY USA; 5 Emeritus Professor of Mathematics, Department of Mathematics, California State University, Fullerton, CA USA

**Keywords:** Predictive markers, Eye manifestations

## Abstract

This report evaluates the role of the combined visual abilities of acuity, contrast sensitivity and presentation time on plate discipline and baseball batting performance. A visual function test (EVTS) was performed on 585 professional baseball players. The results were compared to several common plate-discipline measures. The EVTS test provides a single measure combining target size, contrast and presentation time. Correlations (statistically significant) were found between this measure and several plate discipline metrics (InzoneSwingPct, inzoneFbSwingPct, ChasePct, FbChasePct, BBperPa). Years of major league service did not appear to be related to visual ability. When comparing the best and worst 20% groups based on visual ability, statistically significant improvements ranging from 11.6% in BBperPa to 3.5% in inzoneSwingPct were noted in the better visual function group. Effect sizes ranged from 0.278 to 0.387. These results demonstrate the relationship between basic visual function and batting performance. These are the first results, on a large group at the professional level, to demonstrate this relationship statistically. These results may aid player selection, indicating that batters with better visual function are more likely to be successful when batting and more productive for their team.

## Introduction

It is difficult to imagine sporting success without open eyes and visual information. The precise role of vision in sports performance has been a much-studied topic^1–9^. The general importance of vision in sports was summarized by Zimmerman, Lust and Bullimore^1^ in which they describe the importance of both visual acuity and contrast sensitivity in evaluating the differing visual abilities of athletes and non-athletes. In basketball, researchers noted excellent visual acuity and stereopsis, as well as significant differences from normals^2^. Additionally, shooting success was noted to decrease with reduced visualization of the basket^3^. In soccer, the visual function of English Premier League soccer players was found to be superior to that of normal adults^4^, while the visual tracking ability of Brazilian soccer players was found to be superior in more skilled players as compared to less skilled players^5^. In motorsport, elite academy participants from New Zealand were noted to have superior visual acuity as well as perception time when compared to sex and age-matched controls^6^. In cricket, a sport similar to baseball, Elmurr described^7^ the distinction between “hardware”, the actual visual system; vs. “software”, the processing of visual information. Regan proposed^8^ that cricket batters must predict precisely when and where a pitched ball will be located in order for batting success, while Brenton *et al*. described^9^ the use of electronic glasses to occlude vision soon after the pitcher released the ball, forcing the batter to rely on the prediction of ball movement for batting success.

Hitting a pitched baseball has often been termed the most difficult task in all of sports. In order to have the best chance to hit the baseball successfully, batters describe an ability to identify the spinning seams of the pitched baseball, soon after release of the ball by the pitcher. Additionally, batters often view the placement of the pitcher’s fingers on the baseball, relative to the seams, in order to identify which pitch is being thrown and how it will travel to, and over, home plate. Accurate and timely knowledge of what pitch is thrown as well as its path can assist the batter in planning when and where to place the bat as they swing to hit the ball.

At the professional and elite levels of baseball, pitches can reach top speeds of 107 mph. A relatively slow pitched fastball is often thrown at a speed of 90 mph. At this speed, it takes approximately 400 ms for the baseball to travel from the pitcher to the catcher behind home plate. In general, it takes about 150 ms for the batter to actually swing the bat, leaving 250 ms to see the ball, identify the pitch and make a decision whether to swing or not. Given that a human blink takes approximately 300 ms on average, the batter has less than the blink of an eye to see and decide to swing. Additionally, the baseball is 3 inches in diameter and is being thrown from a distance of about 60 feet making the task of pitch identification even more difficult^10^.

Appreciating that a batter has a very short time to identify a low contrast target which is both very small and is viewed at a great distance, highlights the need for not only exceptionally good visual acuity and contrast sensitivity but also the ability to identify the type of pitch quickly. Standard tests of visual function test visual acuity separately from contrast sensitivity and more importantly present targets for an *unlimited* viewing time. These visual measures have not been shown to relate well to on-field performance metrics in elite baseball players and have not been useful in predicting a specific player’s ability. In fact, a search of the medical literature did not reveal any publications discussing the correlation of standard visual acuity measures and on-field baseball performance in the elite baseball population.

In our previously published work^11^, as well as the work of others^12–14^, the visual function of baseball players as well as other elite athletes has been shown to be superior to that of the general population. To our knowledge, the three parameters of visual acuity, contrast sensitivity and presentation time have not been *combined*, and tested simultaneously, in this population prior to this project.

In an effort to test visual function that more closely resembles the visual needs in baseball batting, we used an iPad-based testing system that combines, and tests simultaneously, the three functions of size, contrast and presentation time^15–17^. Scoring was achieved through a previously performed calibration of each target triplet using a Rasch model (item response theory) which created a unidimensional scoring paradigm from which the CoreScore value is assigned^18,19^. Item response theory is a mathematical procedure which is used to understand the relationship between “latent traits” (in this case target size, target contrast, and presentation time) and an observable measure (in this case a correct or incorrect response). At the conclusion of this procedure, each target is assigned a difficulty score, ranging from +5 to −5. A positive value suggests a target is more difficult than average, while a negative score suggests that the target is easier to properly identify than average. Thus, a zero trait score suggests average difficulty, following calibration of the targets against a cohort representative of the general population.

**Figure 1:**
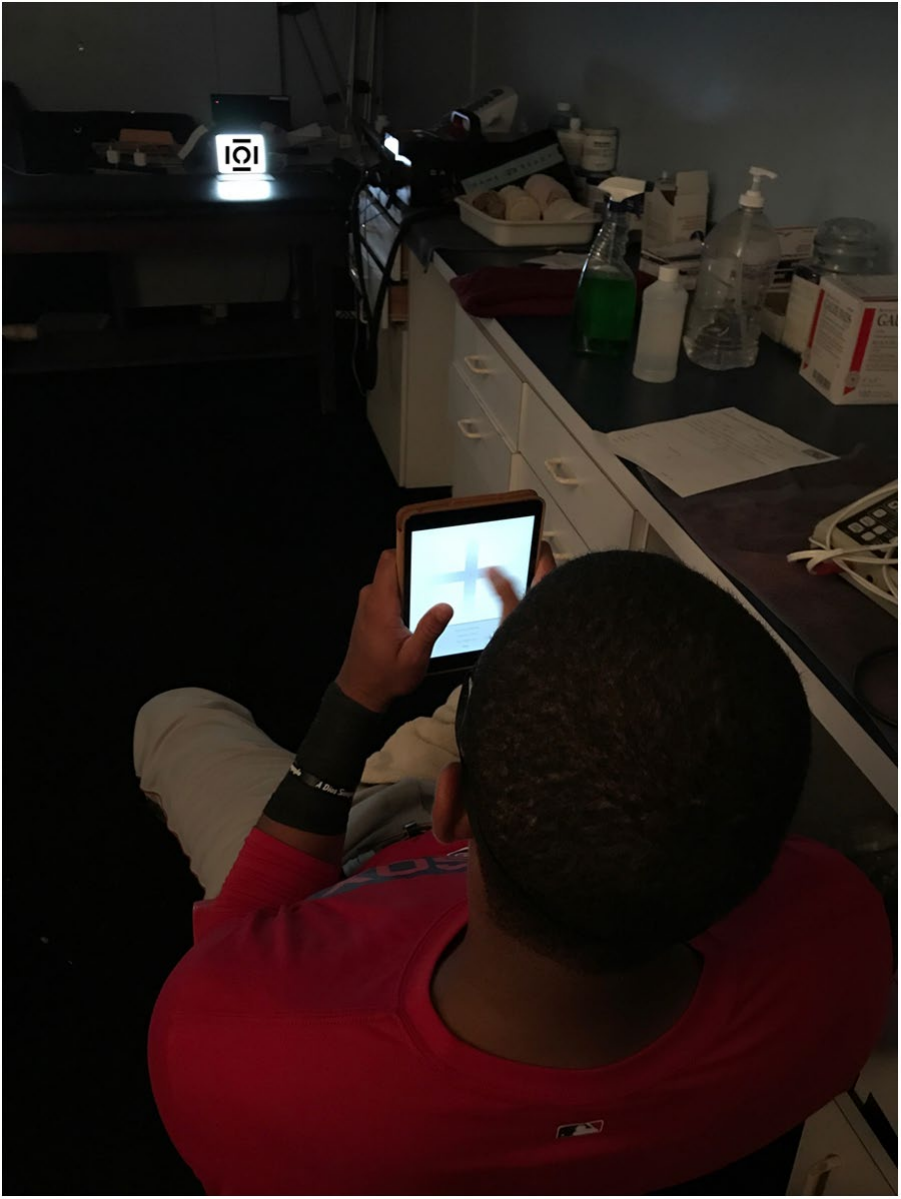
A subject viewing the Landolt ring target on the distant display and identifying the position of the opening by swiping, in that direction, on the hand-held tablet.

In this test, a subject is asked to identify the opening of a Landolt ring in one of four positions (the top, bottom, left or right side of the ring). Each target is composed of a specific size ring (in logMAR units) as well as contrast. Additionally, each target is visible on the display screen for a specified period of time. Thus, each target can be considered a triplet of a specified size, contrast and presentation time (Fig. 1). The difficulty in correctly identifying the opening of the ring will vary based on the target size, target contrast and how long the subject is allowed to view the target. Each triplet had been previously “calibrated” in terms of difficulty, and a step algorithm is used to determine the most difficult triplet the subject is able to reliably identify – which then becomes their score on the test (CoreScore).

In an effort to further understand the relationship as well as the effect of visual ability on baseball batting performance, we undertook this retrospective review of our experience with this test of visual function and very specific measures of batting ability. These measures, termed “plate discipline metrics” rely only on the batter’s ability to judge the pitch and are not influenced by the defense’s ability in the field. Using plate discipline metrics and a vision test that most closely simulates the batter’s viewing conditions, we hoped to gain a greater understanding of the visual needs for baseball batting success.

## Materials and Methods

### Participants

Five hundred and eighty-five professional baseball players were enrolled in this study. A total of 475 subjects had complete plate discipline data (505 subjects for the BBperPA metric) and were included in the analysis. Baseball players were tested during the 2013–2015 baseball spring training camps. For any player that was tested more than once during that period, only their most recent visual results, matching their most recent career batting statistics, were utilized in the analysis. There were one hundred thirty one (132) athletes who were considered major league players (any player who had any major league batting experience). while 373 baseball players were members of the minor league. Length of major league service, on average, for the major league players was (Mean ± Standard Deviation) 3.8 years ± 3.6 years and by definition was 0 years in the minor league player group. Major league players had an average of 381 ± 135 individual at bats per year, the minor league players had an average of 280 ± 133 at bats per year. Player ages ranged from 22 to 43 years. Seventy-four percent of the batters were minor league players and 26% of the cohort were major league players. A total of five professional (Major League Baseball) baseball clubs contributed players to the study and all players were male. This retrospective data review was approved by the State University of New York, College of Optometry, Institutional Review Board (IRB). The study conformed to the tenets of the Declaration of Helsinki. The IRB waived the need to obtain informed consent for this retrospective study per United States Federal Regulation 45 CFR §46.116 (f) (3).

### Materials and design

The EVTS (Enhanced Vision Testing System) is an iPad-based testing system. The iPads were placed four meters apart in a dimly lit room. The display iPad was placed in the landscape position while the controller iPad was held by the subject in the portrait position (Fig. 1). All subjects were tested with their optical prescriptions they used when batting, if any, and were all given the same testing protocol. The subjects were initially tested with the right eye and then the left eye, in isolation while wearing an eye patch. Each test consisted of 122 discrete targets described as triplets consisting of a specific target size, target contrast, and presentation or display time. Landolt rings targets, with crowding bars, were placed centrally on the display iPad. The Landolt rings were oriented with the opening placed on the left, right, top, or bottom of the ring. The subject’s task was to identify the position of the opening and swipe in the direction of the opening (e.g. for an opening on the left, swipe from right to left on the controller iPad).

### Testing procedure

The test protocol, for each eye, consisted of three parts. The first part was intended to acquaint the subject with the testing paradigm. Here, the subject was presented several large targets at 100% contrast which were displayed for an unlimited amount of time. The subject was asked to swipe, on the controller iPad, in the direction of the opening of the Landolt ring. After swiping, another ring was displayed in a random orientation allowing the subject to swipe again. After three consecutive correct swipes, demonstrating an understanding of what was required in the test, the practice portion ended.

Following the initial practice, a second set of practice targets were presented to the subject. In this practice, the triplet targets were more test realistic (i.e. smaller, fainter and displayed for a shorter time) and were intended to acquaint the subject with what was expected during the actual test. In this second practice, a sub-group of 11 targets, taken from the actual testing set, were presented one after the other in sequence. Each subject’s swipe immediately caused the next target to be displayed. The 11 target triplets represented varying target sizes, contrasts and presentation or display times and were not used in scoring.

Following the two practice sessions, the subject then began the actual test using the right eye, with a patch placed over the left eye. The test was comprised of 122 target triplets presented randomly. The subject was instructed to answer each target by swiping in one of the four possible directions, matching the opening of the Landolt ring. Immediately upon swiping, the next target was displayed until the subject reached the end of the testing sequence. Following this, the left eye was tested with a patch placed over the right eye. The testing sequence consisted of the same 122 targets, in a different random order.

Upon completion, the results were transmitted to the analysis software where the subject’s answers were processed and a single value “CoreScore” result was calculated for each eye individually.

The EVTS system has been validated against standard vision testing procedures, both to demonstrate convergent validity as well as to demonstrate test-retest reliability. In an unpublished study, 68 individuals underwent high contrast visual acuity testing, low contrast visual acuity testing, and contrast sensitivity testing at four spatial frequencies (3,6,12, 1nd 18 cpd). The standard Snellen visual acuity of test (ETDRS) subjects ranged from 20/10 to 20/100. Additionally, all testing was repeated 5 days later.

In the convergent validity study, Pearson correlation coefficients, were 0.91 for the high contrast visual acuity testing and 0.94 for the low contrast visual acuity testing (both p < 0.0001). Correlation coefficients for contrast sensitivity testing were noted to be 0.50 at 3 cpd, 0.76 at 6 cpd and 0.72 at 12 cpd and 0.74 at 18 cpd (p < 0.0001). Test re-test results showed a Pearson correlation coefficient of 0.98 when the CoreScore result on the first visit was plotted against the core score result of the second visit. Additionally, Bland-Altman analysis demonstrated no bias, and a high degree of agreement between the two measures. It is possible that other non-visual factors, such as attention, could play a role in the correlation between the two visits.

These results demonstrated good agreement between the EVTS testing approach when compared to standard visual function measures of visual acuity (both high and low contrast) as well as several contrast sensitivity testing levels. In addition, good test-re-test reliability was noted.

Additional details, including the scientific basis on how the test is scored, has been previously published^18,19^.

### Plate discipline metrics

Common measures of baseball batting performance, like “batting average”, “slugging percentage” and “on-base percentage” are unfortunately not purely dependent upon a batter’s skill in putting a baseball into play. The defense can have a significant effect on the result of the live ball – catching it for an out or allowing it to fall to the ground for a hit. Both of these possibilities are unrelated to the batter’s ability and depend on the defense’s ability to successfully react to the hit baseball. Thus, these metrics are not solely reflective of the batter’s ability. Recently, baseball batting metrics have been developed which are more reflective of a batter’s own ability. These new measures have been termed “plate discipline metrics” as they more purely measure only the batter’s ability. The batter must determine which pitches to swing at and those he feels he can successfully hit, or at the same time deciding to not swing at balls outside the strike zone or ones in the strike zone that he determines he won’t successfully put into play^20^.

We chose five plate discipline measures which appeared to be the most relevant to vision.

Plate Discipline Metrics:

- Out of zone chase percentage (ChasePct) –percentage of swings on pitches noted to be outside the strike zone, lower value preferred.
- Fastball chase percentage (FbChasePct) –percentage of swings on fastballs only, that are outside the strike zone, lower value preferred.
- In zone swing percentage (inzoneSwingPct) – percentage of swings at all pitches that are in the strike zone, lower value indicates a more discerning batter.
- In zone fastball swing percentage (inzoneFbSwingPct) – percentage of swings at only fastballs that are in the strike zone, lower value indicates a more discerning batter.
- At bats per base on ball (BBperPA) –number of at bats before a walk is obtained, lower value preferred.

Additional metric:

- Major League Service (YrsMLService) – number of years, in total, in professional baseball (MLB minor and major leagues).

### Statistical method

A Microsoft Excel spreadsheet was used to tabulate all results. For each athlete, career plate discipline metrics were combined with the EVTS data. A player’s lifetime career statistics were used for analysis since they provide the best overall measure of a batter’s skill, minimizing the effect of seasonal fluctuation. The dataset was initially reviewed to determine if the joint distributions of CoreScore and each of the batting metrics follow a bivariate normal distribution. Both the Pearson and D’Agostino tests for normalcy determined that assuming a normal distribution in some cases was not justified, and thus a non-parametric statistical analysis was performed. AnalystSoft Inc., StatPlus:mac - statistical analysis program for Mac OS. Version v5, Minitab Version 14, and SAS version 9.4, Python v3.0 using Google CoLab were used to perform the analysis. Utilizing the entire data set to determine quintiles, the top 20% and bottom 20% of subjects on the CoreScore were compared using the Mann-Whitney test. Additionally, the effect size relating to visual function was calculated using the Hedges’ G method as sample sizes of each of the quintiles were not identical. This allows an assessment of the differences between the top and bottom quintiles of subjects on each batting metric in the units of a joint standard deviation.

## Results

### Normal values for professional baseball players

As the CoreScore results followed a normal distribution, testing results from the right eye and left eye were compared using a paired, 2-tail, student t-test. No difference between an individual’s right and left eye was found (p = 0.363), and thus only results from testing with the right eye were used in this analysis. Batters are trained to view the pitcher with both eyes by turning their face appropriately, resulting in no difference in viewing for right or left-handed batters.

Descriptive statistical details for the CoreScore results as well as the plate discipline metrics are shown in Table 1. The mean CoreScore for this population was 0.971 with a standard deviation of 0.741. In addition, the entire cohort of players, on average, had just over 1 year of service at the major league level. Batters gained a walk after approximately 11 plate appearances (1/0.088 = 11.4 at bats per walk). Mean swing rates ranged from 0.643 to 0.646 for balls in the strike zone and 0.301 to 0.264 for balls outside of the strike zone, depending if they were fastballs or not. One would expect the batter to swing more frequently at strikes than at balls. Additionally, fastballs outside the strike zone were the least frequently swung at pitches, while swing rates at any pitch within the strike zone were similar.

**Table 1:** Descriptive statistical details for the CoreScore results, as well as the plate discipline metrics, for the entire MLB cohort.

	N	Mean	StDev	Median	Minimum	Maximum
CoreScore	585	0.971	0.741	1.000	−1.745	3.041
inzoneSwingPct	475	0.643	0.067	0.649	0.419	1.000
inzoneFbSwingPct	475	0.646	0.077	0.650	0.364	1.000
ChasePct	475	0.301	0.072	0.301	0.000	0.647
FbChasePct	475	0.264	0.073	0.260	0.000	0.571
BBperPA	505	0.088	0.035	0.087	0.000	0.250
YrsMLService	580	1.022	2.452	0.000	0.000	18.000

**Figure 2:**
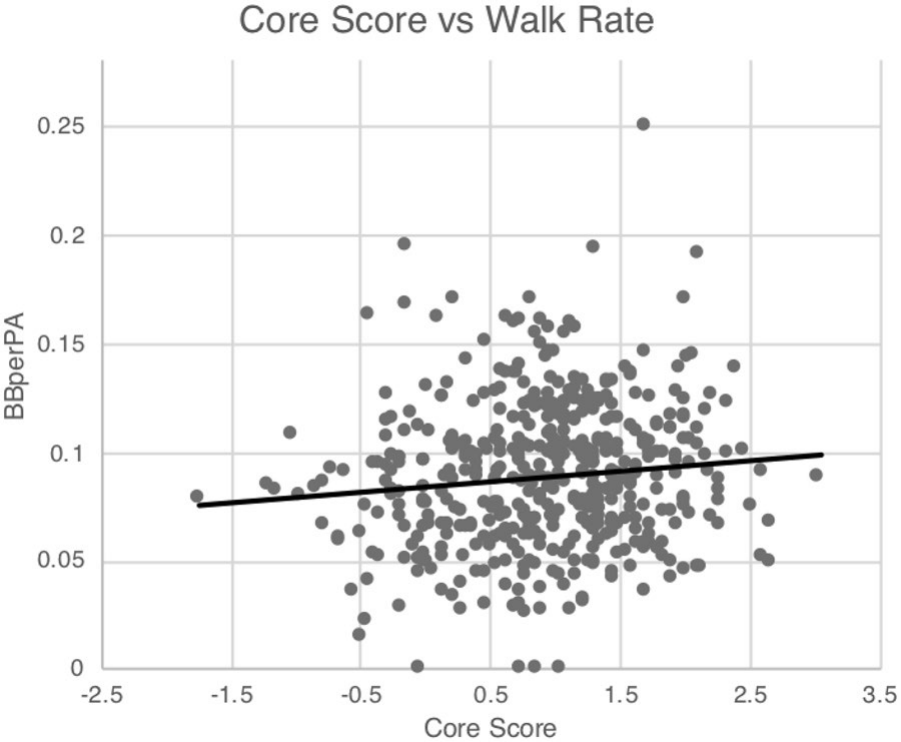
Scatter plot of CoreScore vs BBperPA, with best fit linear trendline. Note that as visual ability increases, so does the walk rate.

**Figure 3:**
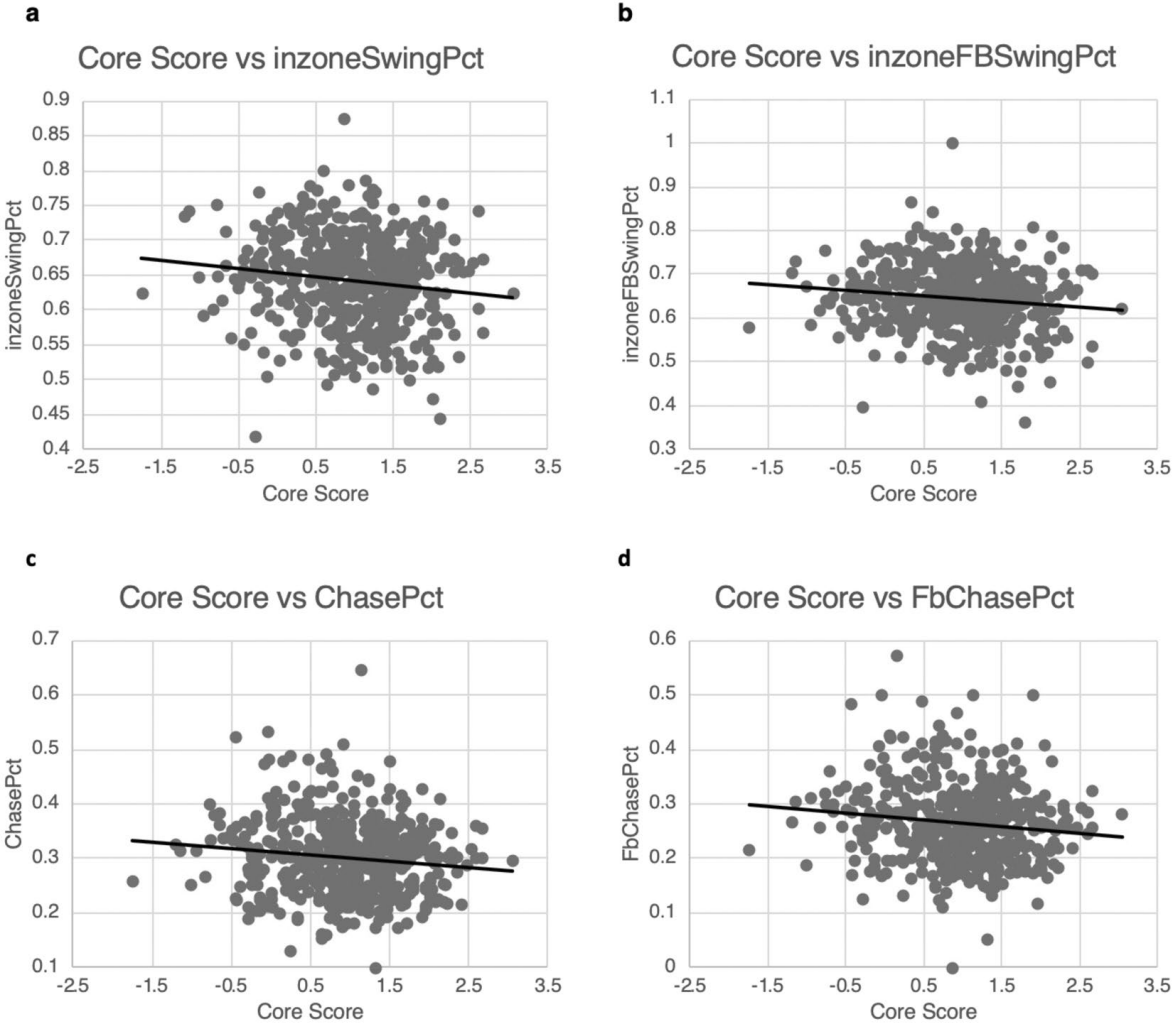
Scatter plot with best fit linear trendline for CoreScore vs InZoneSwingPct, InZoneFbSwingPct, ChasePct, and FbChasePct. Note that in each plot, as visual ability increases as evidenced by larger CoreScore values, the plate discipline metrics decrease indicating greater selectivity in swing decision.

Figures 2, 3 depict graphically the relationship of each of the plate discipline metrics to CoreScore. Examination of the scatter plot, and best fit line of CoreScore versus BBperPA (Fig. 2) demonstrates that as visual ability increases (larger CoreScore values), an increase in walk rate (larger BBperPA values) is observed. Figure 3 contains scatter plots for CoreScore versus each of the remaining plate discipline metrics. In this case, the trendline has a negative slope indicating that with better visual function, the batters are more selective in their swings for both pitches in the strike zone, as well as outside the strike zone regardless if a fastball or not.

Table 2 contains the Spearman correlation results for the CoreScore and plate discipline metrics. Significant statistical correlations ranged from 0.904 for in-zone swing percentage and in-zone fastball swing percentage, to 0.090 for CoreScore and BBperPA. Relatively high correlations were noted between similar plate discipline metrics (e.g. chase and fastball chase percentages as well as in the strike zone swing percentage and the same metric for fastballs alone). Although statistically significant, correlations between the CoreScore and the plate discipline metrics were low, ranging from 0.090 to −0.147. No statistically significant correlation was found between the CoreScore and the number of years of major league service.

**Table 2:** Spearman correlation results for the CoreScore and plate discipline metrics.

	CoreScore	inzoneSwingPct	inzoneFbSwingPct	ChasePct	FbChasePct	BBperPA
inzoneSwingPct	−0.147					
	0.001					
inzoneFbSwingPct	−0.138	0.904				
	0.003	<0.0001				
ChasePct	−0.114	0.430	0.322			
	0.013	<0.0001	<0.0001			
FbChasePct	−0.125	0.359	0.280	0.862		
	0.006	<0.0001	<0.0001	<0.0001		
BBperPA	0.090	−0.385	−0.285	−0.755	−0.655	
	0.043	<0.0001	<0.0001	<0.0001	<0.0001	
YrsMLService	0.056	0.039	0.029	−0.061	−0.050	−0.027
	0.224	0.396	0.522	0.183	0.282	0.555

### The relationship between visual ability and on-field performance

In order to compare the visually best batters to the visually worst batters, the entire CoreScore database was sorted from best to worst. The CoreScores were then divided into quintiles and the top 20% of visually excellent athletes were compared to the bottom 20% of athletes in each of the plate discipline metrics (Table 3). Sorted by CoreScore, the top 20% (Quintile 5) had an average CoreScore of 1.95, while the bottom 20% (Quintile 1) group’s average CoreScore was −0.140.

**Table 3:** The plate discipline ability of the baseball players with the best visual ability (top 20% of CoreScores) as compared to those with the worst visual ability (bottom 20% of CoreScores).

	CoreScore	inzoneSwingPct	inzoneFbSwingPct	ChasePct	FbChasePct	BBperPA	YrsMLService
Mean Top 20%	1.950	0.632	0.629	0.293	0.259	0.091	0.898
StdDev Top 20%	0.290	0.066	0.078	0.057	0.063	0.037	2.110
Mann Whitney (p-value)	—	0.015	0.011	0.015	0.004	0.030	0.480
Mean Bottom 20%	−0.140	0.653	0.654	0.318	0.286	0.081	0.718
StdDev Bottom 20%	0.370	0.062	0.065	0.077	0.076	0.035	1.790
Hedges G	—	0.328	0.348	0.370	0.387	0.278	—

Additionally, the plate discipline ability of the baseball players with the best visual ability (top 20% of CoreScores) was compared to those with the worst visual ability (bottom 20% of CoreScores). Table 3 describes this comparison. For each plate discipline metric, the mean, standard deviation, and a measure of statistical significance (*p* value derived from Mann-Whitney analysis) is noted. Statistically significant differences were found between players with excellent visual ability and those with poor visual ability, at the *p* < 0.05 level. The differences were found to be between 3% and 12%. The difference in walk rate (at bats per base on balls) was the largest with a 12% decrease in the number of at bats taken before a walk in those players with excellent visual ability. As an example, the walks per at-bat metric was larger in the athletes who scored in the top 20% of visual ability (0.091 +/− 0.037 or 10.989 at-bats per walk) as compared to the number of walks per at-bat in the bottom 20% (0.081 +/− 0.035 or 12.346 at-bats per walk) visual ability group (*p* = 0.030).

Having performed 5 statistical evaluations involving the plate discipline metric (1 vision test × 5 plate discipline metrics) a Bonferroni correction for Type I error may be considered. With this approach, only *p*-values less than 0.05/5 (0.01) can be considered significant statistically. Using this stricter definition, the relationship between fbChasePct and visual ability remained statistically significant. The relationship between the in-zone plate discipline metrics and visual ability are of borderline statistical significance at this stricter level of analysis.

Although the correlations comparing the core score to plate discipline metrics were statistically significant and not likely to have occurred by chance and the comparison of the best visual function group to the worst visual function group also yielded differences unlikely due to chance, it is important to assess whether the magnitude of the differences, or the effect of vision, are of importance. Table 3 details the result of effect size calculation for each plater discipline metric. As noted, effect sizes ranged from 0.278 to 0.387 for the plate discipline metrics.

**Figure 4:**
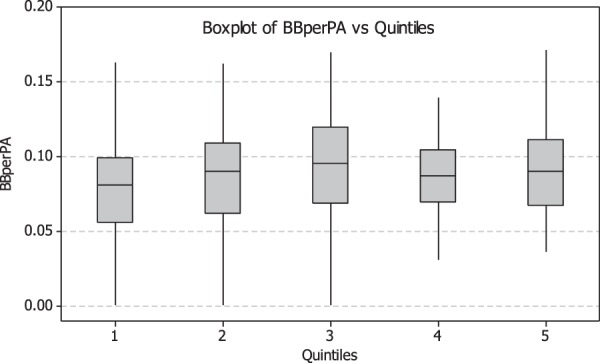
CoreScore quintile plotted against walk rate (BBperPA), with standard error bars. The figure demonstrates an increase in walk rate with increasing visual ability

Figure 4 shows parallel boxplots of walk rate vs. visual ability as described by CoreScore quintiles (20% groups). Quintile 1 represents the worst vison group and has the lowest walk rate (median = 0.080), while Quintile 5 contains those batters with the best vision and the highest walk rate (median = 0.090). Observation of quintiles 2 through 4 in the figure shows them to be approximately equal in their median walk rates (0.090, 0.095, and 0.087respectively). There was a statistically significant difference between the walk rate for Quintile 1 versus those for Quintiles 3,4 and 5 (p = 0.007, 0.040, and 0.030 respectively determined using the Mann-Whitney Test). There was no statistical difference between all other quintile combinations.

**Figure 5:**
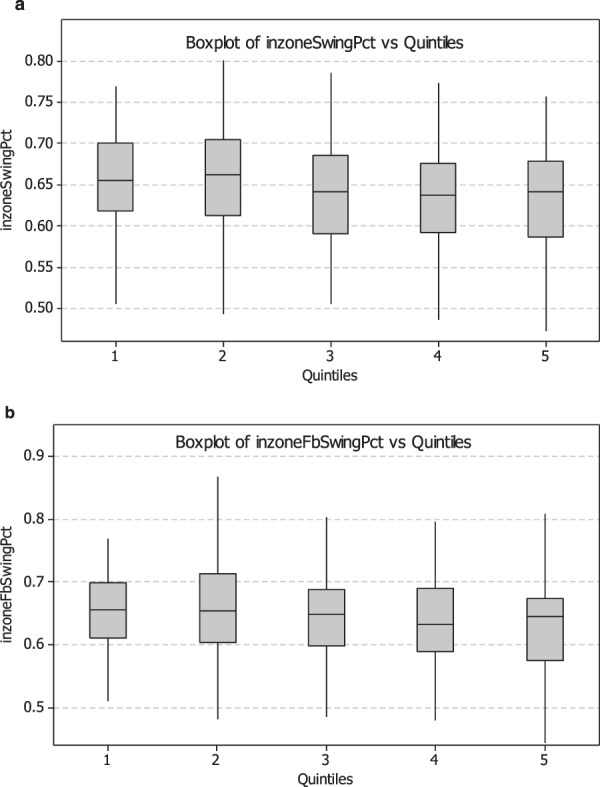
CoreScore quintile, with standard error bars, plotted against in zone swing percentage (**a**) of all pitches (inzoneSwingPct), as well as only fastballs (inzoneFbSwingPct), (**b**) The figure demonstrates a greater likelihood to swing at pitches in the strike zone with *decreasing* visual ability.

Figure 5 shows similar parallel boxplots of in zone swing percentage by visual ability again using the CoreScore quintiles, Fig. 5a; as well as in zone fastball swing percentage (inzoneFbSingPct) Fig. 5b. In this comparison there is an increase in swing rate with reduced visual ability. Batters in the worst vision group (Quintile 1) swung more often than batters in the best vision group (Quintile 5) for both fastballs only (median = 0.655 vs 0.645) as well as all pitches in the strike zone (median = 0.655 vs 0.642). Comparison of the quintiles reveals a statistical difference between Quintile 1 and Quintiles 4 and 5 in Fig. 3a (0.016 and 0.036, determined using the Mann-Whitney test) and between the same Quintiles in Fig. 3b (0.059 and 0.026, again from the Mann-Whitney test).

**Figure 6:**
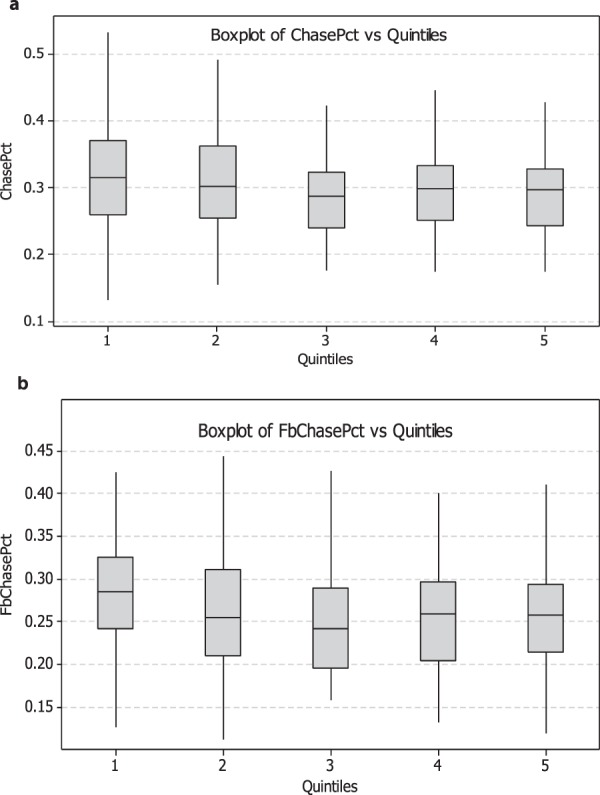
CoreScore quintile, with standard error bars, plotted against chase rate of all pitches (ChasePct), (**a**) as well as only fastballs (FbChasePct) outside the strike zone, (**b**) The figure demonstrates increased swing frequency with decreased visual ability.

Figure 6 show parallel boxplots of chase percentage (ChasePct) versus visual ability (CoreScore) quintiles, Fig. 6a; and fastball chase percentage (FbChasePct), Fig. 6b. The chase rate of batters swinging at pitches outside of the strike zone increased with reduced visual ability (CoreScore quintile). Statistically significant differences were noted between the chase rates of the worst vision group (Quintile 1, median = 0.315) versus the better vision groups (Quintiles 3,4, and 5, medians = 0.287, 0.298, 0.298, respectively) for all pitches outside the strike zone. Statistically significant differences were also noted between the fastball chase rates of the worst vision group (Quintile 1, median = 0.285) versus the better vision groups (Quintiles 3,4, and 5, medians = 0.242, 0.259, 0.258, respectively, p = 0.005, 0.048, and 0.015 for all pitches, and <0.001, 0.010 and 0.004 for fastballs only, respectively).

Evaluation of any possible difference between batters in the major league vs the minor league was also investigated. Seventy-four percent of the batters did not have any major league experience, while 26% had at least some batting experience in the major league. When comparing the CoreScore as well as the plate discipline metrics between the two leagues we found that there was no statistically significant difference between the populations. The minor league mean CoreScore was 0.924 (SD = 0.742), the major league mean was 1.023 (SD = 0.738). A two-sample t-test showed that these two groups were not different (p = 0.185).

## Discussion

This manuscript describes the use of a novel test of visual function that combines several visual abilities that are classically tested independently. With the addition of short and limited viewing times, this test attempts to simulate the viewing conditions present during baseball batting.

Through the use of item response theory^15–17^, the EVTS visual testing system calculates a single vision metric which has been termed the CoreScore. The average result for the general population is set statistically at a CoreScore of zero, thus indicating that the mean CoreScore of 0.971 for professional baseball players noted in this report represents better visual function than normal. This agrees with a previous report^21^ using the same testing system on a group of 1007 professional baseball players. In that report an average CoreScore of 0.820 was noted for professional baseball players comprised of both major and minor league players.

In addition, to confirming previous reports that professional baseball players have better visual ability than the general population, this analysis describes a specific relationship between visual ability and on-field batting performance. By utilizing batter centric plate discipline metrics, instead of the more common measures of batting average, on-base percentage and slugging percentage, we were able to quantify the relationship between vision and batting as well as suggest possible applications of this information. Previous studies have noted relationships between visual reaction time^22^, hand-eye coordination^22,23^, and general vision training^24^ with on-field performance; but no reports associating visual acuity with on-field batting performance in the professional baseball population could be found.

This report demonstrates the relationship between the basic visual functions of size resolution, contrast sensitivity, and limited viewing time with on-field batting measures dependent almost exclusively on the batter’s ability alone without contamination by the abilities of other players on the field. Significant differences between the batters with the best visual ability, as compared to batters with the worst visual ability are noted in the frequency of gaining a walk, swinging at pitches within the strike zone and swinging at pitches outside the strike zone.

We were interested in identifying any difference in batting ability, as measured by the plate discipline metrics, between batters with superior vision and those with poor vision. The decision to divide the dataset into quintiles was based upon the desire to preserve a sufficient number of batters in each group while still allowing sufficient differentiation to be meaningful. Dividing the dataset into 3 groups appeared to be too coarse while dividing the dataset into deciles created too few batters in each group. As noted above, there were statistically significant differences between the worst vision group (quintile 1, bottom 20%) and not only the best vision group (quintile 5, top 20%) but also often with quintiles 3 and 4 (middle 40% of batters) as well on several batting metrics.

The concept of a threshold visual ability as it relates to on-field performance has been suggested in the medical literature in both cricket as well as basketball. Mann, *et al*.^25^ studied the effect of purposefully blurring a cricket batter and noted that up to a certain amount of blur (a +3.00 diopter over correction) there was no effect on batting performance in that sport. In Basketball, Bulson *et al*.^26^ performed a similar study and noted that only with a very strong blurring lens (+10.00 diopter over correction) was there a reduction in basketball free throw ability. Both of these reports suggest that perfect vision is not required for performance of the sports task, and that as long as an athlete has the needed minimum level of vision, they are able to achieve success.

In the current study, review of Figs 4 through 6 suggests a possible threshold relationship, in regard to visual ability and batting performance. Noting the statistically significant differences in each plate discipline metric between those batters in the worst vision group and the better vision group(s), it appears that a certain minimum level of visual ability is required for better plate discipline ability, and as long as that vision is achieved, there may be no statistical difference in batting performance. If a batter’s visual ability is reduced below that minimum level, then batting performance might suffer. Certainly, batters with visual abilities in the worst quintile have reduced ability, but in some plate discipline metrics batters in the 3^rd^, 4^th^, or 5^th^ Quintiles also had significant differences in batting performance compared to the worse vision groups. At this stage, this concept is solely qualitative and theoretical, and awaits further evaluation through additional study.

Although these measures of plate discipline may seem somewhat esoteric, they have a real and material impact on a team’s ability to win games. For example, a batter who gains a walk not only saves his team from recording an out but obtains first base and is thus more likely to score a run for the team (run expectancy theory suggests that an unintentional walk is worth 0.33 runs^20^). Additionally Fig. 6 indicates that batters with poorer vision are less discerning and swing at more pitches outside of the strike zone than batters with better vision. Swinging at pitches outside the strike zone generally leads to an out, either by missing completely or placing the ball weakly into play, leading to an out recorded by one of the fielders. Refraining from swinging at pitches outside the strike zone provides the best chance to gain 1^st^ base through a walk.

What is the actual, on-field, effect of superior visual ability? Using walk rate (BBperPA) as an example, our data demonstrates that those batters whose visual ability is in the best 20% had a walk rate of 0.091 while those in the worst 20% group had a walk rate of 0.081. Assuming that the average batter has approximately 550 at-bats a season, the better vision batters will walk 50.05 times a season (550 × 0.091) while the poorer vision batters will walk 44.55 times each season (550 × 0.081), for a difference of 5.5 walks per season. As noted above, each walk a batter obtains is worth approximately 0.33 runs, resulting in the better vision batter contributing 16.5 runs to his team, while the poorer vision batter contributes only 14.7 runs – a 1.8 run difference for each batter with good vision on the team. This could add up to 16 or more runs per team, per season (9 batters × 1.8), if all batters had better vision as compared to worse vision. Relating this to the popular *wins above replacement* baseball metric, assuming that 10 runs is worth a single win^27^, having a team of batters with excellent visual ability could be worth an additional 1.6 wins per season, simply based on vision. Considering the importance of home-field advantage as well as the number of very close division races in baseball, an additional couple of wins could mean the difference between advancing to the World Series and an early start to the off-season.

Interestingly, batters with better vision also swung less frequently at pitches within the strike zone, allowing a strike to be recorded. Although this appears to be counter-intuitive, further analysis explains why this might be an advantage for batters with good visual function. Albert^11^, in his chapter discussing plate discipline metrics in batters, makes several interesting observations. Firstly, he notes an inverse relationship between batters’ swing rates and contact rates. Specifically, batters who swung often made less contact with the ball and were more likely to strike out, while batters who swung less often were more likely to make contact and place the ball into play. Additionally, he notes that batters who have low swing rates, tended to have higher walk rates (our best visual group) as well as players with high swing rates tended to have lower walk rates (our poorer visual group). Thus, it appears that superior visual ability allows the batter to me more discerning and careful, only swinging at pitches that he is sure he can hit, and not swinging at pitches that he may not successfully place into play.

In addition to demonstrating statistical significance, and a low likelihood that the findings could simply be due to chance, it is important to calculate the magnitude of the difference on batting performance attributed to visual ability^28^. To accomplish this, we calculated the effect size of superior vs. poor visual function (using the Hedges’ G method) on each plate discipline metric. The effect sizes for the plate discipline metrics ranged from small in the case of the walk rate to medium in all other metrics. A not surprising very large effect was noted in the CoreScore calculation as this was the variable used to create the quintiles. Additionally, given the fact that visual ability is only one of many abilities that affect batting performance, it was encouraging to note the medium effect of vision on all but one of the plate discipline metrics.

When comparing the batting statistics of players between leagues of professional baseball, it is important to consider the possible effect of league difficulty on those metrics. Although it is certainly true that many baseball batting metrics can be affected by those opposing a given batter, we purposefully chose plate discipline metrics that were completely, or almost completely, independent of the opposition, being dependent almost exclusively on the batter’s ability. For example, the inZoneSwingPct is completely dependent on the batter’s ability to determine if the pitch is a ball or strike. This metric does not depend on the level of skill of the pitcher, or any other player on the field, it simply depends on the batter’s ability to determine where the pitch will cross the plate and if he chooses to swing or not.

In fact, a recent essay^29^ tracked batters as they progressed through the various minor league levels. They compared the batter’s walk rate (BBperPA) in each level and determined correlations between the A and A+ levels, the A+ to AA levels, and the AA to AAA levels. They found that the walk rates at each stage correlated well suggesting that a batter’s walk rate does not change significantly as they progress through minor league baseball. Additionally, the authors note a fair amount of spread of the data with some batters walking more frequently in the lower levels and others walking more in the higher minor league levels. Finally, all batters are different. Some batters are “power hitters” to whom pitchers often throw around resulting in more walks while other batters are “contact hitters”, who tend to walk less. These findings suggest that by choosing metrics that are highly specific for the batter and are minimally if at all dependent on the opposition, we can minimize any effect of league level on this vision analysis. For additional analysis see Supplemental Information.

Our findings did not show a relationship between visual ability and length of major league service. One would expect that the batters with better vision would have had longer careers, due to greater batting success. Our data did not find a statistically significant difference in career length between the best visual function batters and the worst visual function batters. Although this may actually be the case, it is possible that the relatively small number of major league players in comparison to the greater number of minor league players might have affected the sensitivity of this analysis. Additional studies in the future will be needed to more thoroughly evaluate this possibility.

We also investigated any difference between minor league players and players who had spent at least some time in the major leagues. Similar to the above, there was no statistically significant difference between these groups when compared en masse. This may indeed be a true finding or may be affected by the much larger cohort of minor league players (74% of the cohort) as compared to major league players (26% of the cohort). Additional study will be required to further evaluate this relationship.

Fortunately, some of these visual functions are correctable and trainable leading to improved baseball performance. In a study of collegiate baseball players, Deveau, Ozer and Seitz^30^ described their experience with a perceptual learning task to improve visual acuity in nineteen members of the University of California Riverside baseball team. They noted that players in the trained group showed “impressive improvement in visual acuity” as compared to the non-trained group. Additionally, they evaluated the effect of better visual acuity on on-field baseball performance. Although they did not use plate discipline metrics, they did find a decrease in strike outs, and an increase in the “runs created” metric which they felt was responsible for an estimated 4-5 additional wins for the team during the 2013 season.

These results highlight the importance of using “real world” visual function test to evaluate the visual ability of athletes and determine the relationship to on-field performance. Using this testing system may allow individual athletes, as well as teams, to gauge potential batting ability and take appropriate actions based on that result^31,32^.

## Supplementary information

League level and plate discipline

## Data Availability

The datasets generated during and/or analyzed during the current study are not publicly available due to being comprised of protected health information (United States law 45 CFR Part 46, Subpart A) but are available from the corresponding author on reasonable request.

## References

[1] Zimmerman A, Lust K, Bullimore M (2011). Visual acuity and contrast sensitivity testing for sports vision. Eye Contact Lens..

[2] Quintana S, Roman R, Calco L, Molinuevo S (2007). Perceptual visual skills in young highly skilled basketball players. Percept Mot Skills..

[3] De Oliveira R, Huys R, Oudejans R, van de Langenberg R, Beek P (2007). Basketball jump shooting is controlled online by vision. Exp Pyschology..

[4] Roberts J, Strudwick A, Bennett S (2017). Visual function of English premier league soccer players. Science and Medicine in Football..

[5] Alves M, Spaniol F, Erichsen O (2015). Visual skills of elite Brazilian football players. Eur J Sport Sci..

[6] Schneiders A (2010). Visual acuity in young elite motorsport athletes: a preliminary report. Phys Ther Sport..

[7] Elmurr P (2011). The relationship of vision and skilled movement – a general review using cricket batting. Eye Contact Lens..

[8] Regan D (2012). Vision and cricket. Ophthalmic Physiol Opt..

[9] Brenton J, Muller S, Rhodes R, Finch B (2018). Automated vision occlusion-timing instrument for perception-action research. Behav Res Methods..

[10] Higuchi T (2016). Contribution of visual information about ball trajectory to baseball hitting accuracy. PLoS ONE..

[11] Laby DM (1996). The visual function of professional baseball players. Am J Ophthalmol..

[12] Klemish D (2018). Visual abilities distinguish pitchers from hitters in professional baseball. J Sports Sci..

[13] Hoffman LG, Polan G, Powell J (1984). The relationship of contrast sensitivity functions to sports vision. J Am Optom Assoc..

[14] Stine CD, Arterburn MR, SternN. S (1982). Vision and sports: A review of the literature. J Am Optom Assoc..

[15] Meuse, P. A., Devenport, J. N., Kirschen, D. G. & Laby, D. M. Adaptive visual performance testing system *United States Patent Number US8534839*. Retrieved from http://patft.uspto.gov/netacgi/nph-Parser?Sect1=PTO2&Sect2=HITOFF&p=1&u=%2Fnetahtml%2FPTO%2Fsearch-bool.html&r=1&f=G&l=50&co1=AND&d=PTXT&s1=8,534,839.PN.&OS=PN/8,534,839&RS=PN/8,534,839 (2013).

[16] Meuse, P. A., Devenport, J. N., Kirschen, D. G. & Laby, D. M. Adaptive visual performance testing system *United States Patent Number US8864312*. Retrieved from, http://patft.uspto.gov/netacgi/nph-Parser?Sect1=PTO2&Sect2=HITOFF&p=1&u=%2Fnetahtml%2FPTO%2Fsearch-bool.html&r=1&f=G&l=50&co1=AND&d=PTXT&s1=8,864,312.PN.&OS=PN/8,864,312&RS=PN/8,864,312 (2014).

[17] Kirschen, D. G., Laby, D. M. Methods and systems for intelligent visual function assessments. *United States Patent Number US8967809*. Retrieved from, http://patft.uspto.gov/netacgi/nph-Parser?Sect1=PTO2&Sect2=HITOFF&p=1&u=%2Fnetahtml%2FPTO%2Fsearch-bool.html&r=1&f=G&l=50&co1=AND&d=PTXT&s1=8,967,809.PN.&OS=PN/8,967,809&RS=PN/8,967,809 (2015).

[18] Massof, R. W., Schmidt, K. M., Laby, D. M., Kirschen, D. & Meadows, D. Merging psychophysical and psychometric theory to estimate global visual state measures from forced-choices. *Journal of Physics*. *Conference Series* **459** (1), 012027 (2013).

[19] Laby DM, Kirschen D, Meadows D, Massof RW (2014). A test of visual function combining size, contrast and presentation time. Investigative Ophthalmology & Visual Science..

[20] Albert, J. *Visualizing Baseball*. pp 43, 83-95 (CRC Press, 2018).

[21] Kirschen D, Laby DM, Meadows D (2014). A novel way to assess visual performance in elite athletes. Investigative Ophthalmology & Visual Science..

[22] Burris K (2018). Sensorimotor abilities predict on-field performance in professional baseball. Scientific Reports..

[23] Laby DM, Kirschen DG, Govindarajulu U, DeLand P (2018). The hand-eye coordination of professional baseball players: The relationship to batting. Optometry and Vision Science..

[24] Clark JF, Ellis JK, Bench J, Khoury J, Graman P (2012). High-performance vision training improves batting statistics for University of Cincinnati baseball players. PloS One..

[25] Mann D, Ho N, DeSouza N, Watson D, Taylor S (2007). Is optimal vision required for the successful execution of an interceptive task?. Hum Mov Sci..

[26] Bulson R, Ciuffreda K, Hayes J, Ludlam D (2015). Effect of retinal defocus on basketball free throw shooting performance. Clin Exp Optom..

[27] Slowinski, S. Converting runs to wins. https://library.fangraphs.com/misc/war/converting-runs-to-wins/ (2010).

[28] Sullivan GM, Feinn R (2012). Using effect size – or why the P value is not enough. J Grad Med Educ..

[29] Datadidit. Modeling Walk Rate Between Minor League Levels, https://community.fangraphs.com/modeling-walk-rate-between-minor-league-levels/ (2016)

[30] Deveau J, Ozer D, Seitz A (2014). Improved vision and on field performance in baseball through perceptual learning. Curr Biol..

[31] Drum B, Calogero D, Rorer E (2007). Assessment of visual performance in the evaluation of new medical products. Drug Discovery Today. Technologies..

[32] Classé JG (1997). Association between visual reaction time and batting, fielding, and earned run averages among players of the southern baseball league.”. Journal of the American Optometric Association..

