# Supplementary material for: The Effect of Visual Function on the Batting Performance of Professional Baseball Players
Source: Sci Rep. 2019 Nov 14;9:16847. doi: 10.1038/s41598-019-52546-2 (PMC6856529; doi:10.1038/s41598-019-52546-2)
Supplement: Supplementary file 1 — League level and plate discipline [file 41598_2019_52546_MOESM1_ESM.docx]

Supplemental Information:

The Effect of Visual Function on the

Batting Performance of Professional Baseball Players

* Daniel M. Laby, MD ^1,^

David G. Kirschen, OD, PhD ^2, 3^

Usha Govindarajulu, PhD ^4^

Paul DeLand, PhD ^5^

1. SportsVisionNYC, New York, NY, drlaby@sportsvision.nyc
2. Southern California College of Optometry, Marshall Ketchum University, Fullerton, CA. [davidkirschen@mac.com](mailto:davidkirschen@mac.com)
3. Jules Stein Eye Institute, University of California at Los Angeles, Los Angeles, CA
4. College of Optometry, State University of New York, New York, NY. [ugovindarajulu@sunyopt.edu](mailto:ugovindarajulu@sunyopt.edu)
5. Emeritus Professor of Mathematics, Department of Mathematics, California State University, Fullerton, CA. deland@Exchange.fullerton.edu

Correspondence: Daniel M. Laby MD, Director, SportsVisionNYC, 411 East 57^th^ Street, New York, NY, 10022, tel: 508-507-0158, email: drlaby@sportsvision.nyc

**The effect of League level on Plate Discipline Metrics**

In order to address the effect of league level on our analysis, we returned to the original data and included the league for each batter in the analysis. In this analysis, “0” represents a major league level batter, “1” represents a AAA level batter, “2” represents a AA level batter, “3” represents an advanced single A level batter, “4” represents a single A full season level batter, and “5” represents a short season single A level batter. Thus, the higher the level, the less advanced the batter.

Overall, descriptive statistics for this data set are noted below (Table S1). Note that the N is different than the manuscript data set due to unavailable level (mostly) or at-bat data.

Table S1

We included the number of at-bats for each batter in order to provide a metric that would clearly be related to a batter’s league level. It is expected that batters who are more advanced, toward the major league level, would have experienced a greater number of at-bats than a batter who was only starting in professional baseball at the single A level.

Below is a scatter plot (Fig S1), and a plot of means with 95% CI for the Mean at bats (Fig S2), with best fit lines, for this comparison.


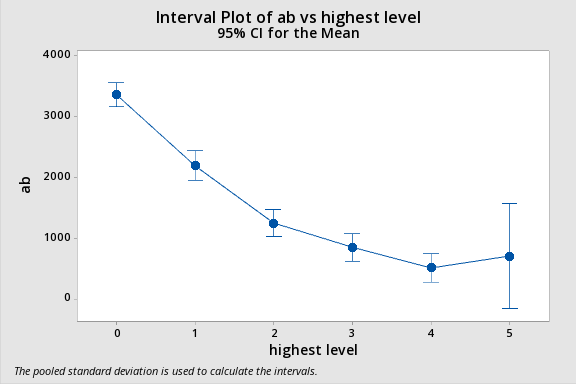

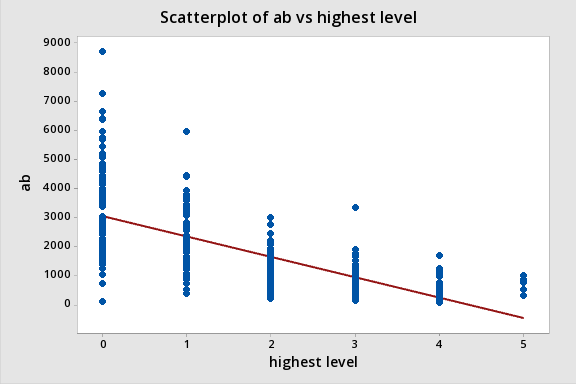
Figure S1 Figure S2

ANOVA evaluation

Table S2

These results (Table S2) suggest that the level of the batter *is* a significant factor in determining the total number of at-bats he has gained. There is a clear distinction between Major league batters, AAA batters, and AA batters. At bats at the A+, and A levels also seem to follow a similar pattern while batters at the A- level are less clearly different likely due to the small N (only 5 batters at this level).

Having demonstrated what was expected in terms of number of at-bats and league level, we now turn our attention to the question “Does league level play a role in these on-field batting measure?”. In order to evaluate, we compared ChasePct to highest level for each of the above batters.

Following is a scatter plot of ChasePct vs Highest level, with best fit line included:

Figure S3


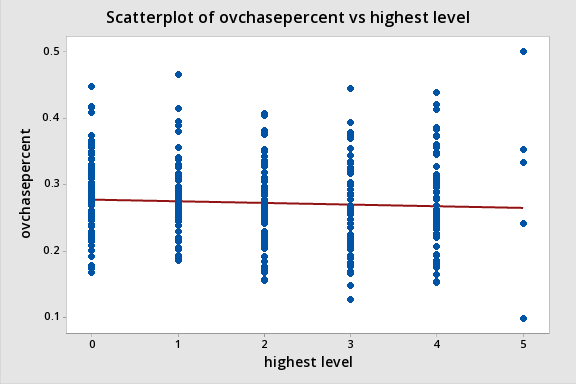


Review of the scatter plot (Fig S3) and Mean plot (Fig S4) shows a fairly flat relationship between ChasePct and the 6 league levels

Table S3


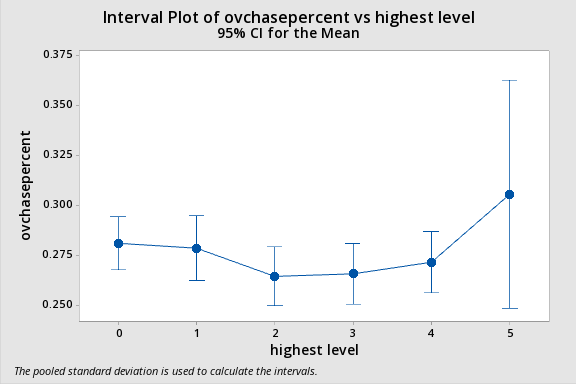
Figure S4

ANOVA evaluation

Table S4

These data suggest (Table S4) that ChasePct is not related to league level, and that batters in the Major League *do not* swing more frequently at pitches outside the strike zone than batters in lower league levels.

In terms of the other plate discipline metrics as they relate to league level:

1. FbChasePct: group 0 was different than groups 3 and 4; groups 2 and 5 could not be distinguished from the other groups.
2. inZoneSwingPct: groups 0 and 2 were different than groups 3 and 4, while groups 1 and 5 could not be distinguished from the other groups.
3. InZoneFbSwingPct: Group 0 was different from groups 3 and 4, which were also different from groups 1 and 2, group 5 could not be distinguished from the other groups.
4. BBperPA: None of the groups could be distinguished from each other

If there indeed was a relationship between league level and the above 5 plate discipline metrics, we would expect to have seen a consistent relationship either across the batting metrics or at least consistently between different league levels (e.g. Major league vs AAA, or major league vs AA, etc.) – which is not the case. Thus, we conclude that these metrics, including ChasePct, are not related to league level as noted in the manuscript.

In addition to the resource already provided in the manuscript on this topic, Below are several additional resources (along with their web citations) that note the consistency of the plate discipline metrics, and their independence of league level (it is important to remember that in some regards the AAA level is a resource pool for the major leagues, and the AA level is the make or break point for many aspiring major league baseball players).

<https://tht.fangraphs.com/improvements-in-plate-discipline-rare-but-effective/>

Kiley McDaniel recently noted that hitters rarely improve their plate discipline after their first three years as a pro, and that hitters who played year-round in high school tend to top out much earlier. The data seem to back up McDaniel’s claims. As research done by Bill Petti demonstrated, none of the core plate discipline metrics vary by more than 3% over the course of a player’s career.

<https://blogs.fangraphs.com/scouting-the-hit-tool-pt-2/>

I did a study for one of the clubs I worked for and the basic takeaway was that, among above average regulars in the big leagues, less than 10% of them materially improved or regressed their plate discipline numbers once they got into pro ball and the more accurate number is probably around 5%. I think much of it is genetic and tied to vision, but how much can be taught, how early it can be taught and if “eye skills” can be taught or better utilized is still very fuzzy and with plenty of exceptions for every supposed rule.

Thus, plate discipline metrics appear stable across most, if not all, league levels and minimally related to factors beyond the batter’s ability. The data in this report does not support a difference in plate discipline metrics based on league level.
